# Oxidative Stress in Dry Eye Disease: Molecular Mechanisms and Emerging Therapeutic Strategies

**DOI:** 10.3390/biom16050718

**Published:** 2026-05-13

**Authors:** Tingting Tang, Jiaxin Yang, Hongbo Yin

**Affiliations:** 1 Department of Ophthalmology, West China Hospital, Sichuan University, Chengdu 610041, China; tangtingting9615@gmail.com (T.T.); yangjiaxin_@stu.scu.edu.cn (J.Y.); 2 West China College of Medicine, Sichuan University, Chengdu 610041, China

**Keywords:** oxidative stress, dry eye disease, reactive oxygen species, antioxidant treatments, nanozymes

## Abstract

Dry eye disease (DED) is a chronic inflammatory disorder of the ocular surface, characterized by tear film homeostasis imbalance, with aging being identified as a crucial independent risk factor. Oxidative stress, which refers to the excessive production of reactive oxygen species (ROS) and reactive nitrogen substances during mitochondrial metabolism and the weakened protective effect of antioxidants, plays a central role in this process. With aging, the mitochondrial function of ocular surface tissues, such as the corneal epithelium, meibomian glands, and lacrimal glands, declines. Concurrently, the activity of endogenous antioxidant enzymes (such as superoxide dismutase and glutathione peroxidase) decreases, and the levels of tear antioxidants such as lactoferrin also decrease. These age-related changes collectively lead to excessive accumulation of ROS, triggering oxidative stress that directly damages biomacromolecules in ocular surface cells and impairs the stability of the tear film. Furthermore, we have summarized the current therapeutic strategies for oxidative stress in DED, including both conventional antioxidants and emerging approaches such as eye drops based on nanoenzymes, thermosensitive hydrogels, intense pulsed light therapy, and drug-eluting contact lenses. By combining the new progress in the delivery systems of biomaterials-based drugs with mechanism-guided interventions, this review systematically establishes the intimate functional linkages between mitochondrial dysfunction, oxidative stress, and the pathogenesis of DED and focuses on elaborating the translational potential of advanced biomaterials-based antioxidant regimens, aiming to provide novel foundations and insights theoretical for the development of more effective and precise therapeutic strategies for DED.

## 1. Introduction

Dry eye disease (DED) is a multifactorial disease of tears and the ocular surface, characterized by tear film instability and potential ocular surface damage. Individuals with DED typically experience symptoms such as foreign body sensation, dryness, itching and burning, which can lead to visual disturbances and fatigue, thereby profoundly affecting their daily life [1]. The global prevalence of DED was estimated to be approximately 11.59% [2], and it is becoming one of the most common ocular surface diseases. This requires more attention from ophthalmologists. Age is a significant risk factor for DED. Large-scale epidemiological studies have shown that the prevalence of DED increases every five years after the age of 50 [3,4,5]. This phenomenon is of great significance, as aging is associated with increased oxidative stress [6], which is in turn linked to chronic inflammation [7]. With age, the concentration of endogenous antioxidants decreases significantly, while the production of reactive oxygen species (ROS) increases [8]. In DED, normal lacrimal secretion is followed by excessive evaporation of the tear film, leading to tear hyperosmolarity. This hyperosmolarity disrupts electrolyte balance, which can damage the ocular surface and tear film, thereby further exacerbating hyperosmolarity and inducing oxidative stress [9]. As a consequence, the interaction of these pathways propagates DED into a vicious circle [10]. Notably, more and more evidence indicates that mitochondrial dysfunction is the central upstream driving factor coordinating ocular surface oxidative stress, cellular damage, and inflammatory cascades, thereby forming a pivotal pathological axis that promotes the progression of DED.

Current therapeutic paradigms prioritize symptom alleviation through ocular surface lubrication and pathomechanistic targeting via anti-inflammatory agents, but they cannot block the pathological processes mediated by oxidative stress and mitochondrial dysfunction. Therefore, breaking the vicious cycle requires a paradigm shift toward innovative interventions that target mitochondrial resilience. Among emerging strategies, antioxidant therapies based on advanced biomaterials have emerged as a cutting-edge approach, offering superior advantages in controlled release, biocompatibility, and precise modulation of the ocular surface microenvironment. Hence, this review aims to provide an integrated and comprehensive summary of the latest advances in DED research. Specifically, it seeks to systematically establish the intimate functional linkages between mitochondrial dysfunction, oxidative stress, and the pathogenesis of DED. Furthermore, this review will focus on elaborating the translational potential of advanced biomaterials-based antioxidant regimens, aiming to provide novel insights and theoretical foundations for the development of more effective and precise therapeutic strategies for DED.

## 2. Oxidative Stress

### 2.1. Mechanisms of Oxidative Stress

“Oxidative stress” is a term that was first proposed in 1985 by German physician Helmut Sies, referring to the imbalance between the production of oxidants and antioxidant defense systems, which may induce damage to biological systems [11]. This condition arises from the excessive generation of free radicals such as ROS and reactive nitrogen species (RNS) by mitochondrial metabolism and decreased activity of the protective antioxidants [12].

ROS are a group of molecules with high oxygen content and strong reactivity, including superoxide anions (O_2_^−^), hydrogen peroxide (H_2_O_2_), singlet oxygen, and hydroxyl radicals (·OH). These molecules can modify and damage cellular macromolecules, including DNA, proteins, and lipids [13]. O_2_^−^, for example, serve as precursors for other ROS and can trigger inflammatory responses. H_2_O_2_ is a stable ROS that can diffuse across cell membranes; it not only regulates signal transduction but also induces cellular oxidative damage. ·OH generated through the Fenton reaction exhibit extremely high reactivity, which can directly cause lipid peroxidation and DNA damage.

**Figure 1:**
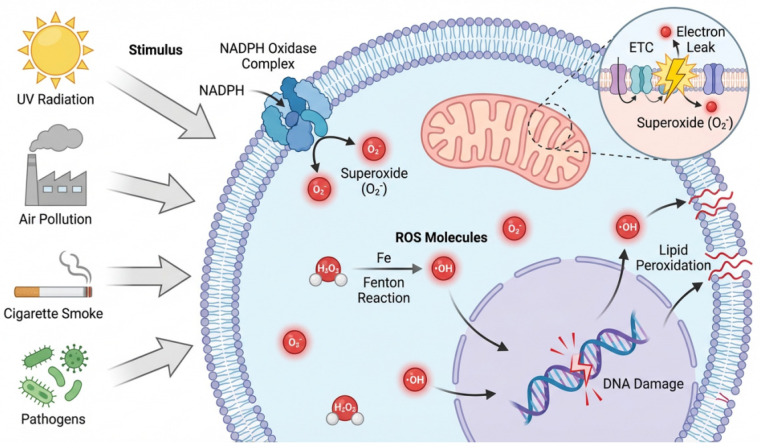
Schematic representation of exogenous and endogenous sources of ROS generation and downstream oxidative damage mechanisms (DNA damage and lipid peroxidation) in eukaryotic cells.

Endogenously, mitochondria are the core site for cellular energy production and also the main source of ROS. ROS are primarily produced within the inner mitochondrial membrane through the electron transport chain, where some electrons leak into oxygen, forming O_2_^−^. Nicotinamide adenine dinucleotide phosphate (NADPH) oxidase (NOX) complex is another important source of O_2_^−^. This complex uses NADPH as an electron donor to convert oxygen molecules into O_2_^−^ [14]. Exogenously, the production of ROS is triggered by exposure to harmful substances, including xenobiotics, pathogens, and ultraviolet radiation. ROS are commonly found in natural water sources, cigarette smoke, air pollutants and other environments [15] and are mainly generated through photolysis and electron transfer processes. Figure 1 illustrates the pathways of ROS generation in eukaryotic cells and the mechanisms of oxidative stress damage.

### 2.2. Biomarkers of Oxidative Stress and Detection

Oxidative stress biomarkers refer to specific biological molecules or indicators utilized for assessing the extent of oxidative stress. In recent years, the detection of oxidative stress biomarkers has attracted increasing attention in the medical field. Clinically validated oxidative stress biomarkers—such as F_2_-isoprostanes (reflecting lipid peroxidation), 8-Hydroxy-2′-deoxyguanosine (8-OHdG, reflecting DNA damage), and the activities of redox enzymes including superoxide dismutase (SOD) and glutathione peroxidase (GPX)—are strongly associated with disease progression and end-stage organ complications [16]. The early detection of their levels is crucial for conducting pathological research, diagnosing diseases, and performing health screenings. Specifically detecting ROS and/or oxidative damage products, as well as evaluating the effects of antioxidants, poses a significant practical challenge. This is because most ROS have a short lifespan (milliseconds or less), low steady-state levels (picomolar to low micromolar range), and rapid fluctuations, which are influenced by continuous changes in production rate, chemical reactions, and diffusion.

Biomarkers related to oxidative stress can be divided into three major categories. The first category involves the direct measurement of reactive species, including O_2_^−^, H_2_O_2_, ·OH, nitric oxide (NO), and ONOO^−^. Due to their extreme instability and short lifespans, detecting these molecules typically requires the use of ultra-sensitive methods, including fluorescent probes, electrochemical methods, or biosensors featuring nanostructured electrodes [17,18]. Currently, electron spin resonance (EPR) and fluorescent probes are the most widely employed techniques for the detection of ROS and RNS. However, EPR is expensive and requires special operating conditions, while fluorescent probes have limitations such as limited specificity, cytotoxicity and insufficient quantification [19,20]. Recent advances in electrochemical biosensing have enabled the detection of ROS and RNS with high sensitivity and practicality. For example, peroxidase-modified electrodes have traditionally been used for the measurement of H_2_O_2_ [20], while the current application of nanomaterials has endowed this detection method with advantages such as high sensitivity and low detection limit [21]. It is worth noting that non-enzymatic H_2_O_2_ sensors based on MnO_2_ nanosheets or platinum nanoparticles can quantitatively detect H_2_O_2_ at micromolar levels in serum in real time [22]. Electrodes modified with NO reductase or hemoglobin have been investigated for NO detection. Recently, carbon electrodes modified with gold nanoparticles can detect NO at nanomolar concentrations in artificial tears [23]. The integration of these electrodes with microelectrodes and lab-on-a-chip platforms further facilitates real-time monitoring of NO released from cells and extracellular fluids [24]. In addition, dual-analyte biosensors have been developed for the simultaneous detection of ROS and RNS. For example, graphene oxide-based electrode capable of simultaneously detecting H_2_O_2_ and NO have been successfully applied in inflammation and ischemia–reperfusion injury models, demonstrating its practicality in monitoring the dynamics of oxidative stress [21]. A review by Mayank [25] systematically summarized elaborates on the unique advantages of 2D materials (such as graphene, TMDs, MXenes) in constructing high-performance biosensors, which enables sensitive and selective detection of various oxidative stress biomarkers, including ROS, RNS, and oxidation damage products.

The second category involves the detection of oxidative damage products caused by ROS and RNS to biomolecules such as lipids and DNA. Researchers widely use high-performance liquid chromatography (HPLC) and mass spectrometry to quantitatively analyze substances such as malondialdehyde (MDA), 4-hydroxynonenal (4-HNE), and isoprostanes, in order to assess the risk of atherosclerosis and diabetic complications [26]. The oxidation of DNA has been extensively studied by measuring the biomarker 8-OHdG in urine and serum to evaluate the risk of chronic diseases and cancer [27]. Electrochemical and optical biosensors targeting oxidative damage products have received considerable attention. A recent study designed a label-free and portable biosensor device that directly detects 8-OHdG via plasma-coupled electrochemistry on transparent and conductive indium tin oxide (ITO) electrodes [28]. This device is a promising biosensor for point-of-care testing (POCT) of 8-OHdG in various biological fluid samples, such as saliva and urine.

The third category focuses on assessing oxidized proteins, such as oxidized albumin and oxidized low-density lipoprotein (LDL) [29]. Although HPLC and mass spectrometry are the gold standards for detecting oxidized albumin, they are limited by high cost and turnaround time in clinical applications. To address these limitations, researchers have developed more efficient detection methods. For example, electrochemical immunosensors with gold nanoparticle-modified electrodes have been used to quantify the oxidation state of albumin within minutes, and have been validated using human serum albumin from patients with kidney disease [30]. Table 1 provides a detailed summary of the oxidative stress biomarkers.

## 3. Oxidative Stress in DED

Recent studies have established oxidative stress as a key link connecting the etiological factors of DED (such as environmental stimuli, meibomian gland dysfunction (MGD) and autoimmunity) and pathological damages (including ocular surface epithelial defects and abnormal neurosensory function) [44]. And central to this process is the associated production of ROS [45]. In normal physiological conditions, the ocular surface regulates physiological antioxidants such as SOD and GPX through the Nuclear factor erythroid 2-related factor 2 (Nrf-2) signaling pathway, thereby maintaining the dynamic balance between the production and scavenging of ROS [46]. However, in DED, ROS is overproduced through three core pathways. The first one is the mitochondrial dysfunction in ocular surface epithelial cells and the leakage of the electron transport chain increases, leading to a 2- to 3-fold higher production of O_2_^−^ than that in healthy individuals [47]. The second one is the imbalance of the meibomian gland margin flora in patients with MGD. Lipases secreted by pathogenic bacteria such as *staphylococcus* decompose the lipids of the meibomian glands to produce free fatty acids, which stimulate neutrophils to release myeloperoxidase. This enzyme then catalyzes the generation of highly oxidizing substances such as hypochlorous acid [48]. The third one is that hyperosmolar tear fluid activates osmoreceptors (such as TRPV1) in ocular surface epithelial cells, triggering an increase in intracellular Ca^2+^ concentration which further activates NADPH oxidase, forming an “osmolarity-ROS” positive feedback loop [49]. Meanwhile, the abnormal maintenance of the tear film leads to a significant decrease in the concentration of antioxidant components such as vitamin C and glutathione in tears. This dual effect disrupts the oxidative balance [50], and excessive ROS can lead to ocular surface damage. Specifically, on the one hand, ROS directly attacks the unsaturated fatty acids in the corneal epithelial cell membrane, triggering lipid peroxidation reactions. This generates toxic products such as 4-HNE and MDA, which damage the integrity of the cell membrane, leading to increased cell permeability and leakage of intracellular contents [51]. On the other hand, ROS oxidatively modifies DNA and proteins, inhibiting signaling pathways related to the proliferation of corneal epithelial cells (such as PI3K-Akt) and delaying wound healing. Clinically, this is manifested as positive corneal fluorescein staining (CFS) [52].

Aging, a well-known trigger of DED, is associated with the increased oxidative stress [53]. Among the various theories of aging, the “free radical theory” has been widely discussed. This theory hypothesizes that aging is associated with structural damage caused by the accumulation of oxidative damage to key macromolecules (lipids, DNA, RNA, and proteins) induced by ROS and RNS [54]. With increasing age, the balance between oxidants and antioxidants is disrupted, leading to the excessive production of free radicals and non-radical substances. The excess of these reactive molecules exerts adverse effects on normal physiological systems [55].

There is a broad consensus that the progression of DED is driven by an inflammatory vicious cycle, a process aggravated by oxidative stress. The activation of the inflammatory cascade is accompanied by the release of inflammatory mediators, which are regarded as tear biomarkers [56]. In a microenvironment characterized by low antioxidant levels, IL-6, a biomarker in tears and conjunctiva, stimulates cells to produce ROS, prostaglandins, and other enzymes [57]. Under such circumstances, an increase in the secretion of cytokines and chemokines will trigger the innate immune response and intensify the inflammatory reaction [56]. Oxidative stress and inflammatory form a closely linked vicious cycle rather than a unidirectional relationship. It has been verified by existing research that ROS can directly activate the transcription factor NF-κB, inducing its translocation from the cytoplasm to the nucleus. Translocated NF-κB then binds to the promoters of pro-inflammatory cytokines such as IL-6, IL-1β, and TNF-α, thereby promoting both their transcription and secretion [58]. This inflammatory response, in turn, exacerbates oxidative stress. Inflammatory cells such as neutrophils and macrophages are recruited to the ocular surface by pro-inflammatory cytokines and release large amounts of ROS, thus perpetuating the cycle. Additionally, under inflammatory conditions, the expression of antioxidant enzymes in ocular surface epithelial cells is inhibited [59], leading to reduced endogenous antioxidant capacity and further amplification of oxidative damage.

In addition to classical apoptosis, novel regulated cell death modalities such as ferroptosis and pyroptosis play important roles in oxidative stress-induced damage in DED. Ferroptosis represents an iron-catalyzed, non-apoptotic cell death that involves three core features: accumulation of lipid peroxides, disruption of iron metabolism, and lowering of GSH and GPX4 levels. These features lead to a reduction in the cellular antioxidant defense capacity and damage to the phospholipid bilayer, ultimately triggering cell death [60,61]. In the corneal epithelium of dry eye model *mice*, the expression of the key regulatory protein GPX4 of ferroptosis is downregulated by 50%, and the content of lipid peroxides is increased by 2.1-fold. Moreover, supplementation with ferroptosis inhibitors like Ferrostatin-1 can significantly improve corneal epithelial integrity [62]. Pyroptosis mediates cell death through inflammasome activation. Under dry eye conditions, mtDNA released from mitochondria damaged by ROS can activate the NLRP3 inflammasome, prompting caspase-1 to cleave gasdermin D. This process forms membrane pores on the cell surface, leading to the release of pro-inflammatory cytokines, as well as cell swelling and lysis [44].

Nrf-2 is the “core switch” for the body to resist oxidative stress and is regarded as a key target in the treatment of DED. Under physiological conditions, Nrf-2 binds to the Keap1 protein in the cytoplasm and remains in an inactive state. When stimulated by ROS, Keap1 undergoes oxidative modification and dissociates from Nrf-2. Translocated Nrf-2 then binds nuclear antioxidant response elements, driving the expression of antioxidant genes like *SOD*, *GPX*, and *HO-1* [46]. Animal experiments have confirmed that in the dry eye model, in a model of sidestream cigarette smoke (SCS) exposure, the tear film break-up time (TBUT) of Nrf-2 gene-knockout *mice* is shorter than that of wild-type *mice*. After SCS exposure, *Nrf2*(-/-) *mice* exhibited markedly elevated fluorescein and Rose Bengal staining scores compared to wild-type *mice* [63]. Clinical studies have also revealed that the nuclear translocation rate of Nrf-2 in the ocular surface epithelial cells of dry eye patients is significantly reduced, and this reduction is negatively correlated with the Ocular Surface Disease Index (OSDI) score (*r* = −0.59, *p* < 0.001) [64]. This finding suggests that the dysfunction of the Nrf-2 pathway is a crucial factor contributing to the imbalance of oxidative stress in DED.

To conclude, oxidative stress runs through the entire process of etiological initiation, pathological injury and inflammatory amplification in DED, forming a core pathological network. Etiological factors including environmental stimuli, meibomian gland dysfunction, hyperosmolar tear fluid and aging trigger excessive ROS production via three core pathways: mitochondrial dysfunction, dysbiosis of meibomian gland marginal flora, and activation of osmoreceptors. Meanwhile, tear film disruption leads to a remarkable decrease in antioxidants such as vitamin C and glutathione in tears, collectively disrupting the ocular surface redox balance. Excessive ROS induces lipid peroxidation, oxidative modification of DNA and proteins, thereby inhibiting corneal epithelial proliferation and delaying wound healing. ROS also mediates novel types of regulated cell death including ferroptosis and pyroptosis to aggravate ocular surface damage. Furthermore, ROS activates the NF-κB pathway to promote the secretion of pro-inflammatory cytokines, while inflammatory responses recruit inflammatory cells to release more ROS and suppress the expression of antioxidant enzymes, forming an oxidative stress-inflammation vicious cycle. As the central regulator of ocular antioxidant defense, the Nrf-2 pathway exhibits impaired nuclear translocation and downregulated antioxidant gene expression in DED, which further exacerbates oxidative imbalance.

**Figure 2:**
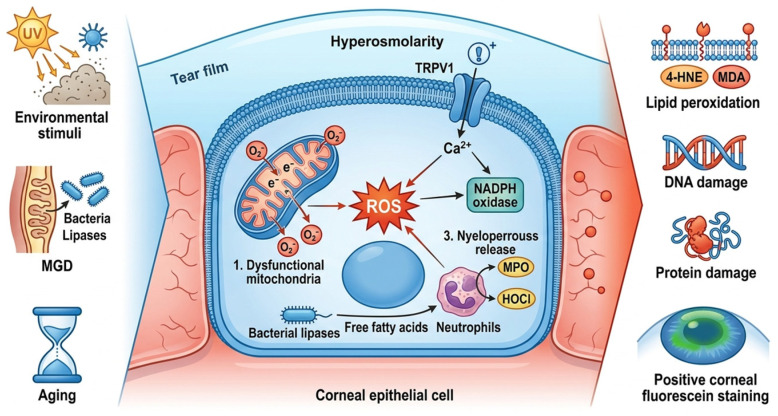
The landscape of oxidative stress in DED.

Collectively, oxidative stress mediates ocular surface injury, regulated cell death and inflammatory cascades through multi-pathway and multi-target effects, serving as a pivotal mechanism in the pathogenesis and progression of DED (Figure 2). Antioxidant treatment has become a key strategy in the treatment of DED.

## 4. Antioxidant Treatments for DED

### 4.1. Systemic Antioxidant Treatments for DED

Oral nutritional supplements have been evaluated as an effective treatment for improving tear film stability and ocular surface epithelium in patients with DED.

Recent years have seen omega-3 polyunsaturated fatty acids, mainly eicosapentaenoic acid (EPA) and docosahexaenoic acid (DHA), draw considerable attention for the clinical management of DED. Their therapeutic effects are mediated by a dual mechanism involving both anti-inflammatory and antioxidant activities. Specifically, EPA competitively inhibits cyclooxygenase and lipoxygenase, thereby reducing the production of pro-inflammatory mediators such as prostaglandin E2 (PGE2) and leukotriene B4 (LTB4). Concurrently, it promotes the synthesis of anti-inflammatory lipid mediators, including resolvin E1. Through this dual action, EPA effectively interrupts the inflammation–oxidation cycle [65]. In contrast, DHA serves as a critical component of cell membrane phospholipids; it enhances membrane fluidity, mitigates oxidative damage to the lipid bilayer, and decreases the level of lipid peroxidation [66]. Several small-scale clinical trials have provided evidence supporting the efficacy of omega-3 fatty acids in DED treatment [67,68,69]. However, the Dry Eye Assessment and Management trial—a large-scale, multi-center, randomized controlled study—demonstrated no significant difference in the mean change in the OSDI score between the active supplement group (n-3 EPA) and the placebo group [70], even though both groups exhibited gradual improvement in symptoms over time.

Astaxanthin is a naturally occurring red carotenoid belonging to the xanthophyll family, which is predominantly found in marine environments. Its unique molecular structure confers important biological properties, mainly characterized by potent antioxidant, anti-inflammatory, and anti-apoptotic activities [71]. Unlike most antioxidants that act only on either the inner (e.g., vitamin E and β-carotene) or outer (e.g., vitamin C) layer of the cell membrane, astaxanthin can penetrate the bilayer membrane. It efficiently exerts antioxidant protection by scavenging ROS in both the inner and outer layers of the cell membrane, with particularly high scavenging activity against ·OH [72]. A single-group clinical study published in 2021 enrolled 60 patients with mild to moderate DED; all participants were instructed to take 6 mg astaxanthin tablets orally twice daily. After one month of treatment, significant improvements were observed in multiple indicators compared with baseline, including OSDI score, non-invasive tear break-up time (NIBUT), tear break-up time (BUT), CFS score, eyelid margin signs, meibomian gland expressibility, meibum quality, and blink frequency [73].

Vitamin D has been increasingly recognized as a nutritional supplement in recent years for its value in DED treatment. Its mechanism of action extends beyond the traditional role of calcium regulation to include immune modulation and antioxidant effects. 1,25-dihydroxyvitamin D [1,25(OH)_2_D], the main active form of vitamin D, can bind to the vitamin D receptor to inhibit the activity of the nuclear factor-κB (NF-κB) pathway and reduce the secretion of pro-inflammatory factors such as interleukin-17A (IL-17A). Meanwhile, it upregulates the expression of Nrf-2 in ocular surface epithelial cells, thereby enhancing endogenous antioxidant capacity [74]. According to an observational study, vitamin D administration via intramuscular injection produced multiple positive outcomes in patients with both vitamin D deficiency and DED that was unresponsive to artificial tears. These included (i) enhanced tear secretion; (ii) reduced tear film instability as well as less inflammation of the ocular surface and eyelid margin; and (iii) alleviated DED symptoms [75].

The beneficial effects of vitamin C in medical prevention and treatment of various diseases have been widely reported, including antioxidant, anti-inflammatory, anticoagulant, and immunomodulatory properties. Vitamin E is a group of fat-soluble compounds with strong antioxidant activity, among which α-tocopherol is the most biologically active isoform. As a major fat-soluble free radical scavenger, it protects cell membranes from lipid peroxidation and oxidative damage by neutralizing ROS and RNS. Early research has shown that supplementation with vitamins C and E in diabetic patients can effectively improve tear secretion and stability, as well as goblet cell density and the degree of squamous metaplasia. This improvement is associated with a significant reduction in NO levels, which may reflect the role of these compounds in alleviating oxidative stress on the ocular surface [76].

A randomized controlled trial investigated the efficacy of an antioxidant supplement containing anthocyanins, astaxanthin, vitamins A, C, and E, as well as various herbal extracts such as *Cassiae semen* and *Ophiopogonis japonicus* in the treatment of DED. Compared with placebo groups, overall subjective impression and objective indicators including tear ROS levels, Schirmer’s test and TBUT significantly improved, speculating that this formulation is effective in increasing tear production and improving tear film stability by reducing tear ROS [77].

### 4.2. Topical Antioxidant Treatments for DED

Traditional artificial tears primarily supplement tear fluid and lubricate the ocular surface. In contrast, novel formulations add antioxidant components to achieve both symptomatic relief and etiological intervention. Currently, the antioxidant components commonly used in clinical practice include osmoprotectants, such as L-carnitine, erythritol, betaine, sorbitol, glycerin, and trehalose, which maintain intracellular osmotic pressure and thereby reduce hyperosmolarity-induced ROS production [78]. Clinical studies have confirmed that L-carnitine combined with erythritol can lower tear osmolarity and alleviate symptoms [79,80], as well as defend cells from oxidative injury [81]. Furthermore, taurine, a sulfur-containing amino acid, directly scavenges ROS and enhances GSH synthesis, thereby safeguarding corneal epithelial cells from oxidative stress [82]. Vitamin E maintains meibomian gland lipid layer integrity via suppression of lipid peroxidation [83].

In addition to clinically commonly used components, some antioxidant components have been verified by basic and clinical studies to have therapeutic effects, and their transformation and application are in the improvement stage. Studies have shown that osmoprotectants (aforementioned) can not only exert symptomatic relief effects, but also reduce the synthesis of matrix metalloproteinases and oxidative stress, as well as regulate the autophagic process [84,85], which further confirms their etiological intervention value. In addition, lipoic acid, as a potential antioxidant component, has been evaluated through experimental studies and may improve tear film stability [86], laying a foundation for its subsequent clinical transformation.

Pterostilbene functionalized graphene nanocomposite (PS-CG) is a novel antioxidant agent for DED, which exerts therapeutic effects via activating the Keap1-Nrf2-ARE signaling pathway to scavenge excessive ROS in corneal epithelial cells. In hyperosmotic stress-induced human corneal epithelial cell (HCEC) models, PS-CG effectively reduces intracellular ROS levels, inhibits cell apoptosis, and decreases lactate dehydrogenase (LDH) release. In benzalkonium chloride (BAC)-induced *mouse* dry eye models, topical administration of PS-CG significantly increases tear secretion, repairs corneal epithelial damage, and maintains the integrity of ocular surface structure [87].

Dual-ROS-scavenging nanozyme-based eye drops (PBnZ) are constructed by exploiting borate-mediated dynamic covalent complexation between n-FeZIF-8 nanozymes (n-Z(Fe)) and poly(vinyl alcohol) (PVA) [88]. PBnZ could eliminate excess ROS through both its multienzyme-like activity and the ROS-scavenging activity of borate bonds. In vitro hyperosmotic DED models and in vivo *mouse* DED models, PBnZ markedly alleviates oxidative stress, suppresses inflammatory cytokine secretion (IL-1β, TNF-α), and ameliorates corneal epithelial defects and tear film instability.

Synthetic porphyrin antioxidant Mn-TM-2-PyP is a potent SOD mimetic that efficiently eliminates superoxide anions and inhibits oxidative stress [89]. In particulate matter-induced *rabbit* DED models and in vitro SIRC cell models, topical Mn-TM-2-PyP significantly reduces CFS, alleviates ocular surface hyperemia, and protects corneal epithelial cells from oxidative cytotoxicity.

Recent studies have explored therapeutic strategies combining agents with complementary mechanisms of action to enhance overall efficacy, which are still in the experimental and preliminary clinical exploration stage, with good application prospects but immature transformation. Antioxidants have been paired with anti-inflammatory agents to stabilize the tear film and improve ocular surface integrity [90,91,92], which provides a new idea for the treatment of DED. In a murine dry eye model, the combination of diquafosol (a mucin secretagogue) and tocopherol (TCP) improved tear film stability, reduced oxidative stress and inflammation, and enhanced conjunctival goblet cell density [93]. In a subsequent study [94] by the same group, eye drops combining 5% Lifitegrast (LF) with TCP demonstrated superior efficacy in improving clinical parameters compared to monotherapy with 0.05% CsA, TCP or 5% LF in a dry eye model. These findings suggest that eye drops integrating anti-inflammatory and anti-oxidative mechanisms offer a promising therapeutic approach for DED, but further clinical verification is needed to promote transformation and application.

### 4.3. Physical Therapy for DED

Among physical therapies for DED, Intense Pulsed Light (IPL) and Vectored Thermal Pulsation (VTP) are the most representative, with IPL having higher clinical application maturity and more sufficient evidence.

IPL has emerged as a core physical therapy for DED in recent years, with high clinical application maturity and clear therapeutic efficacy, especially in evaporative DED associated with MGD. It exerts its therapeutic effects through a triple mechanism of “photothermal effect—anti-inflammatory effect—antioxidant effect”. Specifically, in terms of the mechanism of action, the 500–1200 nm broad-spectrum light emitted by IPL can be selectively absorbed by hemoglobin and melanin in the sebaceous glands of the eyelid margin, generating a local thermal effect. This raises the temperature to a safe range of 40~42 °C, melting the viscous lipids (melting point: 32–38 °C) within the meibomian glands, unblocking obstructed glandular ducts, and reducing the production of lipid breakdown products such as free fatty acids, thereby lowering ROS at the source [95]. In terms of anti-inflammatory and antioxidant effects, IPL can activate the photochemical effects of ocular surface epithelial cells, promoting ATP production and upregulating the nuclear translocation rate of Nrf-2, which in turn enhances the activity of antioxidant enzymes such as SOD and GPX [96]. Moreover, IPL is often combined with microblepharoexfoliation, a procedure that uses a special diamond bur to gently remove the cuticular epithelium and biofilm on the surface of the eyelid margin, thereby reducing ocular surface irritation, further improving therapeutic effects and supported by clinical practice evidence.

VTP is a targeted physical therapy for MGD with relatively clear therapeutic effects but slightly lower clinical popularization and transformation maturity compared to IPL. It achieves the unblocking and evacuation of meibomian gland lipids through the synergistic effect of “heating the inner eyelid and compressing the outer eyelid”, thereby indirectly improving the oxidative environment of the ocular surface [97]. Clinical studies have shown that VTP treatment can increase the patency rate of meibomian gland ducts and reduce the lipid secretion quality score [98], providing reliable evidence for its application in MGD-associated DED.

### 4.4. Novel Topical Therapeutic Technologies Related to Ocular Antioxidant

Novel topical therapeutic technologies for ocular antioxidant delivery focus on efficiently scavenging ROS on the ocular surface and address the key limitation of low bioavailability of traditional drugs through intelligent delivery systems. Among them, nanozyme eye drops (represented by cerium oxide nanoparticles) have relatively sufficient experimental evidence and certain transformation potential; thermosensitive antioxidant hydrogels and caffeic acid-modified gelatin hydrogels are in the exploratory stage with good development prospects.

Cerium oxide nanoparticles (CeO_2_), as a new type of “nanozyme”, possess bidirectional antioxidant properties (capable of scavenging multiple ROS such as O_2_^−^, ·OH, and ONOO^−^ simultaneously) and can function continuously through a regeneration cycle (Ce^3+^/Ce^4+^ valence state conversion), making them a breakthrough technology in the local treatment of DED in recent years with high research maturity and clear therapeutic mechanism [99]. These nanoparticles can mimic the dual enzymatic activities of SOD and catalase, and continuously scavenge various ocular surface ROS (e.g., superoxide anions, hydrogen peroxide) through the Ce^3+^/Ce^4+^ redox cycle without self-consumption, thereby exhibiting regenerative antioxidant properties [99]. Additionally, certain modified cerium oxide nanoparticles (e.g., glycol chitosan-coated cerium oxide nanoparticles, GCCNPs) can upregulate the expression and activity of SOD in ocular surface cells, strengthen the endogenous antioxidant defense system of the ocular surface, and confer long-term antioxidant protection [100]. Regarding ocular surface protection, nanoceria can effectively preserve the morphological integrity of corneal and conjunctival epithelial cells, increase conjunctival goblet cell density, and sustain mucin secretion, thereby stabilizing the tear film, augmenting tear volume, and restoring ocular surface homeostasis [99,100]. Furthermore, nanoceria can alleviate ocular surface inflammation by inhibiting oxidative stress-induced apoptosis and downregulating the expression of pro-inflammatory cytokines (e.g., TNF-α, IL-1β, IL-6), thereby further abrogating the pathological progression of DED [100].

Thermosensitive antioxidant hydrogels, represented by functional cyclodextrin hydrogel (F-CD hydrogel) and resveratrol-loaded sustained-release eye drops, are novel local delivery systems with good application potential. Their therapeutic effects have been verified in cellular experiments, but clinical transformation is still in the exploratory stage. The F-CD hydrogel uses β-cyclodextrin as the backbone and covalently conjugates antioxidant components such as resveratrol (RSV, 3,5,4′-trihydroxy-trans-stilbene) to form a local preparation with “in-situ gelation and sustained release” properties [101]. RSV is increasingly recognized for its strong antioxidant and anti-inflammatory effects in ophthalmic diseases [102], acting through dose-dependent regulation of various signaling pathways. In addition, RSV exerts its antioxidant effect by regulating antioxidant enzymes and blocking free radical damage to DNA [103]. In one study [101], researchers prepared resveratrol-loaded nanoparticles (RSV-NPs) based on acetylated polyethylene glycol-poly (lactic-co-glycolic acid) copolymer (PLGA-PEI) and dispersed them in poloxamer 407 hydrogel, developing a novel eye drop formulation that can achieve sustained release of resveratrol for up to 3 days. This system exhibited significant antioxidant and anti-inflammatory activities in corneal epithelial cells, highlighting its therapeutic potential. Notably, this eye drop formulation is expected to overcome the problem of rapid clearance of existing solution-based formulations, thereby providing a novel drug delivery platform for the treatment of DED and various other diseases associated with inflammation and oxidative stress.

Despite the promising potential of novel antioxidant strategies for DED, clinical translation is hindered by four critical barriers, supported by robust evidence from PubMed-indexed studies. First, safety concerns persist across systemic and topical formulations: oral antioxidant combinations (e.g., anthocyanosides, astaxanthin) may induce subtle systemic effects like reduced diastolic blood pressure, requiring long-term monitoring [104]. Secondly, regulatory pathways remain complex and uncertain: ocular nanomedicines fall into a regulatory gray zone between drugs and combination products, lacking standardized PK/PD profiles and approval guidelines. Thirdly, delivery inefficiencies and modest efficacy limit clinical utility: the ocular surface’s anatomical and physiological barriers (e.g., corneal tight junctions, rapid tear clearance) reduce bioavailability, necessitating frequent dosing and poor patient adherence; conventional antioxidants often exhibit low solubility, poor stability, and monotherapy failure, with combined anti-inflammatory–antioxidant regimens lacking large-scale human validation. Finally, long-term efficacy and translational maturity are unproven: most novel agents (e.g., cerium oxide nanoparticles, thermosensitive hydrogels) remain preclinical, with no Phase III trials, while physical therapies like vectored thermal pulsation lag in clinical adoption compared to intense pulsed light. Collectively, these barriers highlight the urgent need for high-quality long-term clinical studies, optimized nanodelivery systems, and clear regulatory frameworks to accelerate the bench-to-bedside transition of antioxidant therapies for DED.

To systematically summarize the research progress and clinical translational value of antioxidant strategies in the treatment of DED, we integrate and compare four major categories of interventions—systemic nutritional supplements, topical antioxidants, physical therapies, and novel ocular drug delivery systems—into a summary table of antioxidant-related therapies for DED (Table 2).

## 5. Future Research Directions and Prospects

Lacrimal gland cell defects represent a crucial pathological process in aqueous-deficient dry eye and ocular surface diseases. Structural and functional abnormalities directly lead to insufficient tear secretion, decreased tear film stability, and progressive glandular damage, making them a key target for etiological treatment and regenerative medicine research of dry eye [105]. The lacrimal gland is mainly composed of acinar cells, ductal epithelial cells, and myoepithelial cells, and defects in various cell types participate in disease pathogenesis through distinct mechanisms. As the core structure for tear synthesis and secretion, acinar cell atrophy, apoptosis, and secretory dysfunction are prominent features of aqueous-deficient dry eye. Impaired *WNT* signaling inhibits acinar cell proliferation and repair, whereas targeted activation of the *WNT* pathway promotes lacrimal acinar cell regeneration and ameliorates tear deficiency [105]. Meanwhile, aquaporin 5 (*AQP5*) deficiency triggers endoplasmic reticulum stress, ROS accumulation, and NLRP3 inflammasome activation, further aggravating acinar cell pyroptosis and secretory arrest [106,107]. Mitochondrial dysfunction, defective autophagy, and aberrant immune activation in ductal epithelial cells contribute significantly to autoimmune-related lacrimal injury, and PINK1/Parkin-mediated mitophagy deficiency accelerates aging-related lacrimal cell damage [108]. Based on the pathological mechanisms of lacrimal cell defects, targeting signaling pathways such as *WNT* and FGF, restoring *AQP5* expression and mitochondrial homeostasis, and combining stem cell and organoid technologies to reconstruct lacrimal architecture have emerged as important strategies for future precision therapy. These approaches provide experimental and theoretical foundations for moving beyond conventional symptomatic treatment toward functional restoration of the lacrimal gland.

Nanotechnology offers a promising strategy for drug delivery. According to statistics, there are currently about 51 nanomedical products available on the market, some of which have entered the clinical trial phase [109]. For example, Xia et al. developed a novel hierarchical action liposome nanosystem [110]. This nanoparticle overcomes the ocular surface transport barrier through a strategy of “ocular surface electrostatic adhesion-lysosomal site-directed escape”. At the therapeutic level, it achieved mitochondrial targeting and antioxidant effects via the SS-31 peptide, and exerted anti-inflammatory effects by loading insulin to reduce mitochondrial inflammatory metabolites. Ultimately, this system breaks the vicious cycle associated with DED through the synergistic effect of “antiinflammation, antioxidation, and mitochondrial function restoration”.

Contact lenses represent a promising ocular drug delivery system, offering distinct advantages over conventional eye drops. By prolonging the residence time of drugs on the ocular surface, they effectively improve drug bioavailability, enhance patient compliance, mitigate the risks of overdosing and adverse side effects (particularly for chronic ocular diseases such as glaucoma) [111]. As a relatively mature platform for controlled ocular drug delivery, drug-eluting contact lenses have been further optimized through the integration of novel polymeric carriers, enabling sustained drug release. Polymeric nanoparticles or implants can be tailored to match the specific characteristics of target drugs, thereby improving encapsulation efficiency and extending the drug release duration. However, several critical challenges must be addressed prior to clinical translation, including drug leakage during storage and transportation, as well as potential lens surface roughness induced by carrier incorporation [112]. Beyond their role in drug delivery, contact lenses are evolving into multifunctional ocular protection platforms that integrate visual clarity, UV shielding, and antioxidant activity. Chen et al. [113] developed a functionalized nanocomposite contact lens that incorporates all these features; the team adopted a copolymerization strategy to incorporate cationic [2-(methacryloyloxy)ethyl] trimethylammonium chloride (DMC) into traditional gel-like contact lenses, transforming their structure from dense to porous. Through electrostatic interactions, this structural modification significantly enhances the loading capacity of a ROS scavenger, namely dark blue Mo^5+^-based polyoxometalate (POM). Within the optical zone, selective oxidation of Mo^5+^-POM to colorless Mo^6+^-POM produces the DMC-POM-based cosmetic contact lens (DPCCL). This lens maintains commercial-grade transparency while offering superior UV shielding and ROS neutralization ability. During the neutralization process, DPCCL generates oxygen and undergoes a color change, allowing prolonged wear and providing a visual indicator for real-time monitoring of ROS elimination.

Individualized treatment based on multi-omics technologies (such as genomics and proteomics) represents an important direction for the future. Among them, ferroptosis and GPX4 may serve as novel therapeutic targets for DED and other ocular surface diseases. Specifically, decreased expression of GPX4 can lead to the accumulation of lipid ROS, and then promote conjunctival cell death and the development of oxidative stress-related diseases. Studies have shown that although apoptosis is the primary cell death mechanism linked to GPX4 deficiency, ferroptosis, as an identified form of cell death, may also serve as a potential mechanism underlying conjunctival cell alterations and various ocular diseases [114]. Sakai et al. [115] confirmed that GPX4 plays a crucial role in maintaining oxidative homeostasis and protecting cells from cytotoxic damage in human conjunctival epithelial cells.

## 6. Conclusions

In recent years, with the increasing emphasis on geriatrics and preventive health concepts and the development of medical treatment concepts from traditional symptomatic alleviation to a new phase of etiological intervention and precision medicine, antioxidant therapy has been gradually applied to the systemic and local treatment of DED. Despite the promising potential of various antioxidant strategies in the management of DED, current interventions are associated with several notable limitations. First, systemic nutritional supplements show significant heterogeneity in clinical evidence: large randomized controlled trials failed to demonstrate significant advantages of omega-3 fatty acids over placebo, whereas studies on astaxanthin and vitamin D are mostly limited to small sample sizes, non-randomized designs, or observational data without high level evidence. Second, conventional topical antioxidants mainly provide symptomatic relief with mild antioxidant effects; monotherapy with single agents is often insufficient, and novel components such as alpha lipoic acid lack clinical data in humans. Although combined anti-inflammatory and antioxidant regimens exhibit synergistic effects in animal models, they remain at the experimental and early clinical stage without large-scale validation. Thirdly, physical therapies are clinically mature but require professional administration; meanwhile, vectored thermal pulsation is less widely adopted and translationally less mature than intense pulsed light. Finally, novel nano-delivery systems, including cerium oxide nanoparticles and thermosensitive antioxidant hydrogels, demonstrate excellent antioxidant activity and sustained release properties but have not yet been evaluated in large-scale human trials, resulting in immature clinical translation. Collectively, current antioxidant therapies are limited by inconsistent evidence, modest efficacy, delivery constraints, and slow clinical translation, highlighting the need for higher quality clinical studies and optimized formulation technologies. In the future, through a further understanding of the pathogenic mechanism and treatment principle of DED, oxidative stress-targeted therapies will achieve precision and long-term efficacy, thereby providing safer and more effective therapeutic options for patients.

## Figures and Tables

**Table 1:** Oxidative stress biomarkers, associated diseases, and evolution of detection strategies.

Category	Core Biomarkers	Common Detection Methods	Associated Diseases	Latest Detection Technology Advances	References
ROS/RNS	Superoxide anion (O_2_^−^)	Chemiluminescence (luminol-based), spectrophotometry (NBT assay)	Cardiovascular diseases, inflammatory disorders, ischemia–reperfusion injury	Fluorescent probe technology for high-sensitivity in situ imaging; 2D material-based biosensors with enhanced electron mobility	[25,31]
	Hydrogen peroxide (H_2_O_2_)	Chemiluminescence, peroxidase-modified electrodes	Cardiovascular diseases, metabolic syndrome, inflammatory diseases	Co_3_O_4_ nanostructured electrochemical sensors for real-time detection; nanozyme-based sensing with low cost and rapid response	[32,33]
	Nitric oxide (NO)	NO reductase-modified electrodes, gold nanoparticle carbon electrodes	Cardiovascular diseases, diabetic vascular complications, inflammatory bowel disease	Nanomaterial-enhanced electrochemical sensors for trace level quantification	[25,31]
Lipid Peroxidation Markers	Malondialdehyde (MDA)	Thiobarbituric acid (TBA) assay, high-performance liquid chromatography (HPLC)	Cardiovascular diseases, Alzheimer’s disease, metabolic syndrome, diabetes	Standardized colorimetric/fluorescent kits for reduced interference; LC-MS/MS for accurate quantification in complex matrices	[34,35,36]
	4-Hydroxynonenal (4-HNE)	Enzyme-linked immunosorbent assay (ELISA), mass spectrometry (MS)	Neurodegenerative diseases, cancer, cardiovascular diseases, inflammation	Specific antibody-based ELISA for rapid screening; HRMS for precise structural identification	[31,37]
	Oxidized low-density lipoprotein (oxLDL)	Monoclonal antibody-based ELISA, HPLC	Atherosclerosis, cardiovascular diseases, metabolic syndrome	Lipidomic assays for comprehensive oxLDL subfraction analysis; nano-enhanced immunosensors	[38,39]
Nucleic Acid Oxidation Markers	8-Hydroxy-2′-deoxyguanosine (8-OHdG)	ELISA, HPLC, LC-MS/MS	Cancer, diabetes, cardiovascular diseases, Alzheimer’s disease	ELISA with pg/mL-level sensitivity; LC-MS/MS combined with isotope internal standards for high accuracy	[31,37,40]
	8-Hydroxyguanosine (8-OHG)	Mass spectrometry, Western blotting	Acute kidney injury, chronic kidney disease, neurodegenerative diseases	Urine-based non-invasive detection technology optimization; high-resolution sequencing for oxidation site mapping	[31,37]
Protein Oxidation Markers	Protein carbonyls	2,4-Dinitrophenylhydrazine (DNPH) derivatization, HPLC	Aging, cancer, neurodegenerative diseases, diabetes complications	HPLC-UV standardization for quantitative analysis; multiplex detection with modified DNPH reagents	[37,41]
	3-Nitrotyrosine (3-NT)	ELISA, LC-MS/MS	Inflammatory diseases, cardiovascular diseases, neurodegenerative diseases	LC-MS/MS for precise quantification; 2D material-based biosensors for enhanced specificity	[25,31]
	Oxidized albumin	Specific antibody ELISA, gold nanoparticle-modified electrode electrochemical detection	Hypertension, chronic kidney disease, liver disorders, diabetes	Gold nanoparticle-modified electrode electrochemical detection, smartphone-compatible sensors	[42,43]

**Table 2:** A comparative table of antioxidant-related treatments.

Category	Intervention	Mechanism	Evidence Type	Study Model/Population	Key Outcomes	Limitations	Clinical Translation Status
Systemic Antioxidant Therapy	Omega-3 fatty acids (EPA + DHA)	EPA: anti-inflammatory/antioxidant, interrupts inflammation–oxidation cycle [65]; DHA: protects cell membrane [66]	Small-scale trials [67,68,69]; large multicenter RCT [70]	Patients with DED	Small scale: improved tear film; DAM trial: no significant difference vs. placebo	No superior efficacy vs. placebo in large RCT	Routine use, controversial efficacy
	Astaxanthin	Scavenges ROS, potent antioxidant/anti-inflammatory/anti-apoptotic [71,72]	Single-arm clinical study [73]	Patients with mild-moderate DED	Significant improvement in OSDI, NIBUT after 1 month	Small sample, no control, limited evidence	Clinical nutritional supplement stage
	Vitamin D	Inhibits NF-κB pathway, upregulates Nrf-2 [74]	Observational study [75]	Vitamin D deficiency + DED refractory to artificial tears	Promotes tear secretion, reduces inflammation	Non-randomized, lack of long-term follow-up	Clinical adjuvant supplementation
	Vitamin C + Vitamin E	Synergistic antioxidant/anti-inflammatory, reduces NO [76]	Early clinical study [76]	Diabetic patients with DED	Improves tear secretion, tear film and goblet cell density	Limited sample, lack of large-sample verification	Routine clinical nutrition supplementation
	Combined antioxidants	Reduces tear ROS, alleviates oxidative stress [77]	RCT [77]	Patients with DED	Significant improvement in subjective and objective indicators	Complex composition, lack of long-term safety data	Clinical supplementary exploration stage
Topical Antioxidant Therapy	Osmoprotectants	Maintains osmotic pressure, reduces ROS, regulates autophagy [78,79,80,81,82,83,84,85]	Clinical + basic studies [79,80,81,84,85]	In vitro cells, patients with DED	Relieves symptoms, protects cells, reduces inflammation	Single intervention, needs combination for severe DED	Widely used in artificial tears
	Taurine	Scavenges ROS, promotes GSH synthesis [82]	Basic + clinical studies	In vitro cells, animal models, patients	Reduces corneal damage, improves ocular microenvironment	Lack of high-quality large-sample RCTs	Clinical adjuvant agent
	Topical Vitamin E	Inhibits lipid peroxidation, protects meibomian lipid layer [83]	Basic clinical studies	Patients with MGD-related DED	Stabilizes tear film, relieves dryness	Poor solubility and bioavailability	Adjuvant additive in artificial tears
	Lipoic acid	Antioxidant, improves tear film stability [86]	Experimental study [86]	In vitro cells, animal models	Alleviates oxidative stress, improves tear film	No Phase III data, not clinically applied	Preclinical transformation stage
	PS-CG nanocomposite	Activates Keap1-Nrf2-ARE pathway, scavenges ROS [87]	Basic experiment [87]	HCECs, *mouse* DED models	Reduces ROS, repairs cornea, increases tears	Nanomaterial safety unclear, no human trial	Preclinical research stage
	PBnZ nanozyme eye drops	Dual ROS scavenging, inhibits inflammatory factors [88]	Basic experiment [88]	In vitro, *mouse* DED models	Alleviates oxidative stress, improves cornea and tear film	No human trial, delivery system safety to verify	Preclinical exploration stage
	Mn-TM-2-PyP	SOD mimetic, scavenges superoxide anions [89]	Basic experiment [89]	*Rabbit* DED, SIRC cell models	Reduces corneal staining, protects epithelium	No clinical translation data	Preclinical research stage
	Antioxidant + anti-inflammatory combination	Synergistic anti-inflammatory/antioxidant, stabilizes tear film [93,94]	Animal experiments [93,94]	*Mouse* DED models	Superior to monotherapy, improves indicators	Lack of large-scale human RCTs	Experimental and preliminary clinical exploration
Physical Therapy	Intense Pulsed Light (IPL)	Photothermal unblocks meibomian glands + anti-inflammatory/antioxidant [95,96]	Clinical practice + basic research	Evaporative, MGD-related DED patients	Improves meibomian function, reduces oxidative stress	Expensive equipment, operator-dependent	Clinically well-established
	Vectored Thermal Pulsation (VTP)	Heating + compression unblocks meibomian glands [97]	Clinical study [98]	Patients with MGD-related DED	Increases gland patency, improves tear film	Low popularity, limited applicable population	Limited clinical use
Novel Topical Delivery Systems	Cerium oxide nanoparticles (CeO_2_)	Nanozyme with dual antioxidant activity, protects ocular surface [99,100]	Basic experiments [99,100]	In vitro cells, animal models	Maintains epithelial integrity, stabilizes tear film	No Phase III clinical data, safety to verify	High-potential preclinical stage
	Thermosensitive antioxidant hydrogels	In situ gelation + sustained release, long-term efficacy [101,102,103]	Cellular studies [101,102,103]	Corneal epithelial cells	Overcomes rapid clearance of traditional formulations	Only cell verification, no animal/human trials	Exploratory stage

## Data Availability

No new data were created or analyzed in this study. Data sharing is not applicable to this article.
